# Reduced Lateralization of Attention in Action Video Game Players

**DOI:** 10.3389/fpsyg.2019.01631

**Published:** 2019-07-17

**Authors:** Yu Li, Xiaohong Jin, Yuanyuan Wang, Dun Niu

**Affiliations:** ^1^Brain and Mind Institute, The Chinese University of Hong Kong, Hong Kong, China; ^2^Department of Cognitive Science, Macquarie University, Sydney, NSW, Australia; ^3^Student Affairs Office, Wuhan Polytechnic College, Wuhan, China; ^4^College of Education, Qufu Normal University, Shandong, China

**Keywords:** action video game playing, attention, lateralization, executive, reorienting

## Abstract

There is increasing evidence that action video game players (AVGPs) possess superior performance in various tasks, especially those measuring attentional abilities. The current study aimed to examine the lateralization of attentional components in AVGPs. Twenty-nine AVGPs and twenty-six non-AVG players (NAVGPs) were recruited based on their frequency and intensity of playing action video games in the last 6 months. A lateralized attentional network test was used to measure the lateralization of attentional components in the two groups. The results showed that AVGPs exhibited comparable performance in the left and right hemispheres for reorienting and executive components. However, NAVGPs exhibited a significant difference between the two hemispheres for the two components. The findings indicate that AVG playing is closely associated with reduced lateralization of attentional networks.

## Introduction

Increasing studies have revealed that compared to non-action video game players (NAVGPs), action video game players (AVGPs) perform better in various tasks (Powers et al., 2013; Wang et al., 2016; Bediou et al., 2018; Kowal et al., 2018; see Latham et al., 2013 and Green and Bavelier, 2015 for reviews). Attentional ability has been extensively examined in these studies, providing convergent evidence that AVG playing is associated with enhanced attentional capabilities (Green and Bavelier, 2003, 2012; Boot, 2015) and neural changes in the brain (Bavelier et al., 2012; Wu et al., 2012; Tanaka et al., 2013; Kühn et al., 2014; Gong et al., 2016), indicating an experience-based neural plasticity in attentional networks associated with AVG playing (Boot, 2015). The current study aimed to specifically examine the lateralization and efficiency of attentional networks associated with AVG playing.

Attention consists of three basic components, alerting, executive, and orienting (Posner and Boies, 1971; Posner and Petersen, 1990; Petersen and Posner, 2012). Alerting processes are involved in establishing and maintaining a state of sensitivity to surroundings, executive processes resolve cognitive conflicts evoked by multiple, incongruent attentional cues, and orienting processes direct individuals to a specific stimulus (Posner and Petersen, 1990; Corbetta and Shulman, 2002; Fan et al., 2005; Corbetta et al., 2008). A brief computerized battery, the attentional network test (ANT), has already been developed to assess the efficiency of the three components (Fan et al., 2002). In the test, different cue types are implemented to measure the efficiency of attentional components. Specifically, alerting effects are obtained by comparing no-cue with center/double-cue conditions, executive effects are obtained by comparing congruent with incongruent flanker conditions, and orienting effects are obtained by comparing valid-spatial-cue with center/double-cue conditions (Fan et al., 2002). Recent research has provided evidence for the association of AVG playing with the improvement of these attentional functions. Chisholm et al. (2010) and Mishra et al. (2011) found AVGPs outperformed NAVGPs in the suppression of distracting information, Green and Bavelier (2003) found that AVGPs showed better flanker compatibility effects, and Boot et al. (2008) found that AVGPs showed higher efficiency of task-switching. These findings indicate more efficient executive functions in AVGPs. As for orienting functions, it was found that orienting processes evoked by exogenous cues operate similarly in AVGPs and NAVGPs. Using a Posner cueing paradigm where an exogenous cue is briefly presented in one of two possible target locations before the target presented in either the cued location or the uncued location, Castel et al. (2005) found that AVGPs and NAVGPs benefited from the cue comparably, i.e., comparable exogenous orienting processes. Castel et al.’s finding is then supported by later studies that also applied a Posner cueing paradigm (Dye et al., 2009; Hubert-Wallander et al., 2011). But for alerting functions, no significant differences between AVGPs and NAVGPs were found in both children and adults (Dye et al., 2009). To summarize, the existing literature has revealed that extensive AVG playing is highly associated with the improvement of some facets of attention, especially executive component.

It has been established that the human attentional brain network is overall lateralized to the right hemisphere (Corbetta and Shulman, 2002; Corbetta et al., 2008; Vossel et al., 2014). Greene et al. (2008) and Asanowicz et al. (2012) added visual field (VF) factor to the ANT and developed a computerized tool, the lateralized attentional network test (LANT), to evaluate attentional functions in the two hemispheres. In the test, attentional components are obtained by comparing two of the four cue types, no-cue, center/double-cue, valid-cue, and invalid cue (see Fan et al., 2009 for the details). Reorienting effects (also called validity effects) are obtained by comparing invalid-cue with valid-cue conditions (i.e., reorienting to unexpected, but relevant stimuli from pre-cued locations). Disengaging effects are obtained by comparing invalid-cue with center/double-cue conditions (i.e., disengaging from pre-cued locations). The test has been used by later studies to determine the lateralization of the attentional functions (e.g., Marzecová et al., 2013; Spagna et al., 2016, 2018). It was found that in the general population, alerting functions were bilaterally implemented in the brain (Greene et al., 2008; Asanowicz et al., 2012; Spagna et al., 2016, 2018); orienting functions were biased to the right hemisphere (Experiment 1 in Greene et al., 2008; Spagna et al., 2016, 2018); reorienting functions were biased to the right hemisphere (Experiment 2 in Greene et al., 2008; Asanowicz et al., 2012; Spagna et al., 2016; but see Spagna et al., 2018); disengaging functions were bilateral (Spagna et al., 2016, 2018); and executive functions were biased to the right hemisphere (Asanowicz et al., 2012; Marzecová et al., 2013; Spagna et al., 2016, 2018; but see Experiment 2 in Greene et al., 2008). Interestingly, the right-hemisphere bias for reorienting functions observed above is broadly in line with earlier work by Evert et al. (2003), and Evert and Oscar-Berman (2001) who found a VF asymmetry for the invalid-cue condition of the Posner’s cueing task. Collectively, these findings indicate that reorienting and executive functions are right-lateralized, and alerting functions do not show a lateralized pattern. These studies have demonstrated the feasibility and reliability of LANT in detecting the lateralization of attentional networks.

However, the lateralization of attentional networks in AVGPs is still poorly understood. A recent study revealed that AVG playing is associated with reduced response bias to the left VF (Latham et al., 2014). In this study, the authors used a line bisection task in which participants were asked to bisect horizontal lines printed on a paper. Typical middle points of lines bisected by normal right-handers are 2% left to the true middle point (Jewell and McCourt, 2000), reflecting the crucial role of the right hemisphere, especially temporoparietal junction, in visuospatial attention (Thiebaut de Schotten et al., 2011). The right temporoparietal junction is much involved in orienting and reorienting functions (Corbetta et al., 2008; Vossel et al., 2014). Therefore, the reduced leftward bias in AVGPs is more likely to be indicative of the reduced lateralization of visuospatial attention, especially orienting and reorienting functions. This finding could be interpreted by the nature of games. In the games full of competition and cooperation, AVGPs have to vigilantly monitor the computer screen with balanced visual monitoring and make fast, accurate responses to multiple various visual cues. In daily game playing, a bias to the left or right VF could be highly detrimental to performance, which has to be avoided by AVGPs. Thus, the right-lateralized attention observed in the general population would be altered to be more bilateral in AVGPs. However, this is not tested yet.

Using the LANT implementing a Posner cueing paradigm in the left and right VFs, this study aimed to test the lateralization of attentional components and its relationships with AVG playing. To measure the ability of AVGPs to reorient to an unexpected location from an expected location and disengage from an unexpected location, we added validity of spatial location, i.e., 20% of spatial cues were invalid, to the test. Effects of alerting, executive, orienting, reorienting, and disengaging were extracted by comparing different cue types (Fan et al., 2009; Spagna et al., 2018). According to the literature reviewed above, we hypothesized that long-term AVG playing would change the lateralization of attentional networks, more specifically reduce the lateralization of executive and reorienting functions.

## Materials and Methods

### Participants

Twenty-nine AVGPs (mean age, 19.55 years; age range: 18–24 years; 13 females) and twenty-six NAVGPs (mean age, 19.11 years; age range, 18–22 years; 14 females) were recruited from Wuhan Polytechnic College, Wuhan, Hubei, China. Each of them participated in this study voluntarily and obtained no course credits or money. Each of them had normal or corrected-to-normal vision, and had no history of neurological impairments. The games played by AVGPs were but not limited to the following games: *The King of Fighters, Temple Run, Counter-Strike, Crossfire, Overwatch, and Need for Speed*, on their computers, tablets or mobile phones. They played games at least 2 h per day and 4 days per week in the last 6 months or more. The NAVGPs didn’t play any action video games in the last 6 months. This study was approved by the Institutional Review Boards of Qufu Normal University and each participant signed an informed consent form before the experiment.

### The LANT

Building on the experimental paradigms used in two previous studies (Greene et al., 2008; Asanowicz et al., 2012), the present study used a LANT to measure the lateralization of alerting, conflict (i.e., executive), orienting, validity (i.e., reorienting), and disengaging effects in the two groups. Greene et al. (2008) and Asanowicz et al. (2012) have demonstrated the reliability of LANT in measuring the lateralization of attentional networks. This tool has also been used to detect the influences of bilingualism on the asymmetry of the networks (e.g., Tao et al., 2011; Marzecová et al., 2013).

**FIGURE 1:**
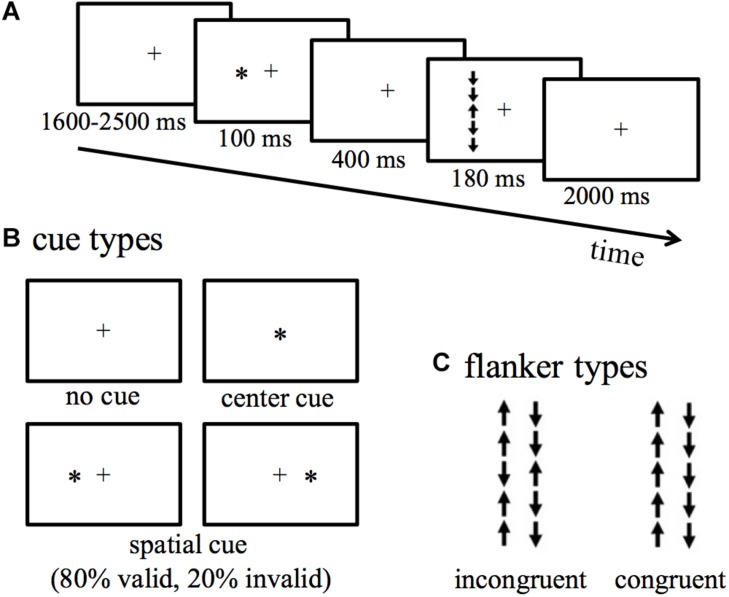
The lateralized attentional network test (LANT). **(A)** Experimental procedure. **(B)** Cue types. **(C)** Flanker types.

The details of the LANT are provided in Figure 1. During this task, a fixation (black cross) was presented in the center of the screen, followed by an up- or down- pointing arrow appeared in the left or right VF with equal probability. The target arrow was flanked by two above arrows and two below arrows with their direction congruent or incongruent with the target arrow. Participants were instructed to indicate the direction of the target arrow by pressing buttons. Reaction times (RT) and accuracy were recorded. The difference in RT and accuracy between incongruent and congruent conditions reflects conflict effects (Fan et al., 2002, 2005). The efficiency of alerting, orienting, validity, and disengaging effects were measured by the differences in RT and accuracy between two of four types of cues. A no-cue is presented before the appearance of the target arrow; a center-cue is presented in the same location as the fixation; a valid-cue is presented in the left or right VF to indicate the location of the target arrow to be presented; an invalid-cue is presented in the left or right VF, but invalidly indicates the location of the target arrow to be presented, and this type of cue appeared 20% spatial cue trials. See the definition of each effect in Section “Data Analyses” below.

### Stimuli and Procedure

The fixation cross was 5 mm (0.47°) in width and 5 mm (0.36°) in length. The target arrow and arrows (flankers) around it were 7 mm (0.73°) in length, and an arrow chain (5 arrows) was thus 35 mm (3.65°). In the task, an arrow chain was presented 60 mm to the left or right VF of the screen. The cue was an asterisk of 5 mm diameter (0.47°) in width and 5 mm (0.36°) in length. The cue was presented in the center of the screen (center-cue), spatially at the same location as the following target arrow (valid-cue), or spatially at the opposite location to the following target arrow (invalid-cue). The distance between the screen and the participants’ eyes was about 60 cm.

The experiment included 432 trials presented in six blocks with 72 trials in each block. In one-half of the trials, the target arrow was flanked by congruent arrows, and in another half flanked by incongruent arrows. Of all the trials, 192 trials were indicated by center-cue or no-cue; 192 trials were indicated by valid-cue presented in the left or right VF; 48 trials were indicated by invalid-cue, which accounts for 20% of all the spatial trials.

The entire procedure was compiled and controlled by E-Prime 2.0 (Psychology Software Tools, Inc., Pittsburgh, PA, United States). For every single trial, a fixation was presented in the center of the screen for varying 1600–2500 ms duration (and the fixation remained through the entire trial), then one of the four types of cue was presented for 100 ms. After the offset of the cue, a blank was displayed for 400 ms, then a target arrow flanked by four arrows with the same or opposite pointing direction appeared for 180 ms. Participants were told that some of the locations of arrows were not predictable (i.e., cues were invalid), but they did not know the ratio (4:1) of valid and invalid conditions. Participants were instructed to indicate the direction of the target arrow by pressing buttons within 2000 ms as quickly and accurately as possible. Participant’s right hand held the mouse of the computer to respond. The mouse was first placed at the middle line to the computer screen and rotated to be parallel to the screen. Participants pressed the right mouse key if the target arrow pointed up and pressed the left mouse key if the target arrow pointed down. This approach enabled participants to respond easily as the direction of the target arrow was spatially compatible with the button response. Before the actual experiment, a separated practice block was given to make participants familiarize with the procedure. Participants were allowed to have a short break between blocks. The entire experiment lasted for about 50 min.

### Data Analyses

For the calculation of the RT and accuracy scores for the attentional networks for each group and VF, we adopted different strategies. For RT, we subtracted the center-cue condition from the no-cue condition to obtain *alerting* scores, subtracted the congruent from the incongruent conditions to obtain *conflict* scores, subtracted the center-cue from the valid-cue conditions to obtain *orienting* scores, subtracted the center-cue from the invalid-cue conditions to obtain *disengaging* scores, and subtracted the valid-cue from the invalid-cue conditions to obtain *validity* scores. Validity is also called reorienting as it reorients attention to unexpected, but behaviorally relevant stimuli. As for accuracy, we used subtractions that were inverse to the subtractions used for RT data to obtain scores. So for both RT and accuracy, a lower score was associated with a higher efficiency. Effects sizes were reported as partial eta squared (η_*p*_^2^). See Fan et al. (2009) for the details of the operational definitions for these effects.

A mixed design was used in this experiment, with *cue type* (no-cue, center-cue, valid-cue, invalid-cue), *congruency* (congruent, incongruent), and *VF* (left, right) as within-subject factors, and *group* (AVGPs, NAVGPs) as between-subject factor. To investigate the interactions between these four factors (e.g., Callejas et al., 2004; Fan et al., 2009) and avoid misinterpreting the results by simply relying on the differences between conditions (Dye et al., 2009), we conducted an omnibus ANOVA, but focused on the significant interactions involving *group* factor. We further conducted simple effects analyses with Bonferroni correction to inspect significant interactions.

## Results

Analyses for RT and accuracy were separately conducted. Trials with RT shorter than 150 ms, longer than 1500 ms (∼1% of correct responses), and trials with incorrect responses (∼15% of all trials) from the RT analysis were excluded from the analyses. Table 1 provides mean RT and accuracy for each condition in the two groups (also see Supplementary Figure S1).

**TABLE 1:** Mean reaction time of trials with correct responses and accuracy for each condition.

			**Accuracy**				**Reaction time (ms)**			
			**AVGP**		**NAVGP**		**AVGP**		**NAVGP**	
Cue	Flanker	VF	mean	SD	mean	SD	mean	SD	mean	SD
No-cue	Con	Left	0.96	0.04	0.92	0.07	456	100	483	92
		Right	0.94	0.06	0.93	0.08	476	105	483	86
	Inc	Left	0.81	0.12	0.78	0.14	521	103	542	97
		Right	0.75	0.15	0.75	0.13	532	108	541	95
Center-cue	Con	Left	0.97	0.05	0.94	0.04	413	100	452	107
		Right	0.95	0.08	0.96	0.05	421	98	438	94
	Inc	Left	0.89	0.10	0.87	0.11	497	107	519	88
		Right	0.85	0.11	0.83	0.10	505	91	524	99
Valid-cue	Con	Left	0.99	0.03	0.97	0.02	331	86	343	83
		Right	0.98	0.03	0.99	0.02	337	105	349	89
	Inc	Left	0.95	0.05	0.97	0.02	389	115	394	88
		Right	0.94	0.04	0.95	0.04	393	112	400	97
Invalid-cue	Con	Left	0.83	0.13	0.85	0.14	520	119	544	124
		Right	0.79	0.17	0.72	0.15	512	111	559	148
	Inc	Left	0.72	0.17	0.66	0.21	570	119	594	149
		Right	0.69	0.19	0.52	0.24	563	127	602	182

*Con, congruent; Inc, incongruent; VF, visual field; AVGP, action video game players; NAVGP, non-action video game players.*

### Overall RT and Accuracy

The mean RTs of correct responses were 465 ms (±98 ms) for AVGPs and 485 ms (±98 ms) for NAVGPs. The mean accuracies were 0.876 (±0.056) for AVGPs and 0.85 (±0.061) for NAVGPs. *T*-tests revealed no significant group difference in both RT [*t*(53) = –0.784, *p* = 0.125] and accuracy [*t*(53) = 1.639, *p* = 0.107]. The accuracy of the right VF stimuli with invalid cue and incongruent flanker was significantly different from chance level (50%) in AVGPs (*p* < 0.001) but not in NAVGPs (*p* = 0.743; see Supplementary Figure S1). The performance of other conditions was significantly higher than chance level in the two groups (*p* < 0.001).

### Alerting Effects

**FIGURE 2:**
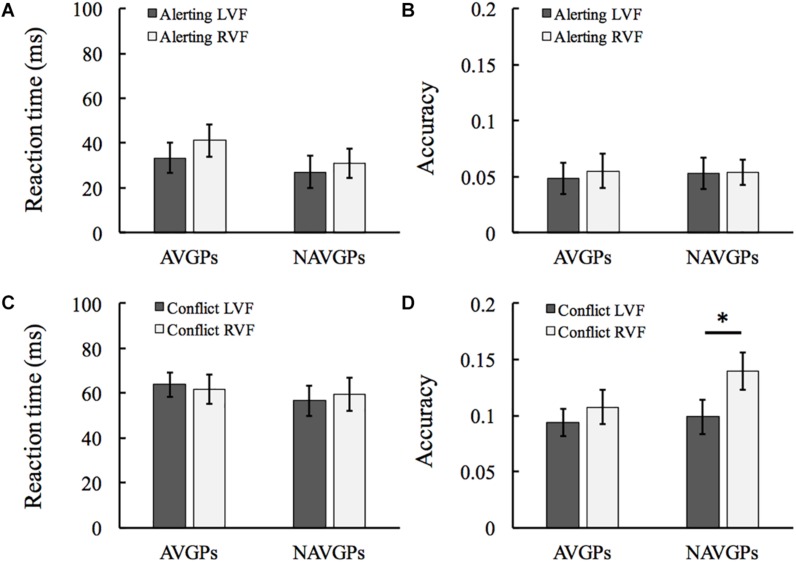
Alerting effects in the left and right visual fields (L/RVF) in the action video game players (AVGPs) and non-action video game players (NAVGPs) **(A,B)**. Conflict effects in the left and right VFs in AVGPs and NAVGPs **(C,D)**. Note that a lower score indicates a higher efficiency for both reaction time and accuracy data. Error bars denote SEM. ^*^p < 0.05.

The difference between no-cue and center-cue conditions was calculated as an index of alerting effects. See Supplementary Table S1 for the mean and standard deviation (SD) of RT and accuracy in the two groups. For RT, a 2 (*group*) × 2 (*VF*) mixed ANOVA revealed that all the *group* and *VF* effects and the interaction effect were not significant [*F*(1,53) < 1.119, *p* > 0.295, η_*p*_^2^ < 0.021; see Figure 2A]. Similarly, all the main effects and the interaction effect were not significant for accuracy [*F*(1,53) < 0.13, *p* > 0.72, η_*p*_^2^ < 0.002; see Figure 2B].

### Conflict Effects

The difference between incongruent and congruent conditions were calculated as an index of conflict resolving (executive function). See Supplementary Table S1 for the mean and SD of RT and accuracy in the two groups. For RT, a 2 (*group*) × 2 (*VF*) mixed ANOVA revealed that all the main effects and the interaction effect were not significant [*F*(1,53) < 0.381, *p* > 0.54, η_*p*_^2^ < 0.007; see Figure 2C]. For accuracy, the *group* and interaction effects were not significant [*F*(1,53) < 1.361, *p* > 0.296, η_*p*_^2^ < 0.025]. The *VF* effect was significant [*F*(1,53) = 5.465, *p* = 0.023, η_*p*_^2^ = 0.093], indicating that the efficiency of right-hemisphere executive effect is higher than the left executive network. Further analyses with *T*-tests revealed a significant difference between the two VFs in NAVGPs [*t*(53) = –2.399, *p* = 0.024], but not in AVGPs [*t*(53) = –1.076, *p* = 0.291; see Figure 2D].

### Orienting Effects

**FIGURE 3:**
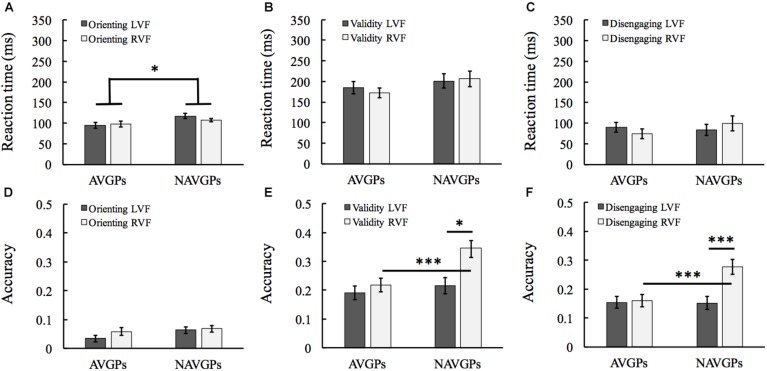
Orienting **(A,D)**, validity (reorienting; **B,E**), and disengaging **(C,F)** effects in left and right visual fields (L/RVF) in the action video game players (AVGPs) and non-action video game players (NAVGPs). Note that a lower score indicates a higher efficiency for both reaction time and accuracy data. Error bars denote SEM. ^*^p < 0.05, ^∗∗∗^*p* < 0.001.

The difference between center-cue and valid-cue conditions were calculated as an index of orienting effects. See Supplementary Table S1 for the mean and SD of RT and accuracy in the two groups. For RT, a 2 (*group*) × 2 (*VF*) mixed ANOVA revealed that the *group* effect was significant [*F*(1,53) = 4.291, *p* = 0.043, η_*p*_^2^ = 0.075; AVGPs, 96 ± 32 ms, NAVPs, 112 ± 22 ms; see Figure 3A]. Detailed inspection revealed that AVGPs had faster responses to the targets preceded by center-cues compared to NAVGPs, but the two groups benefited similarly from valid-cues. For accuracy, a 2 (*group*) × 2 (*VF*) mixed ANOVA revealed that all the effects were not significant [*F*(1,53) < 2.07, *p* > 0.156, η_*p*_^2^ < 0.014; see Figure 3D].

### Validity Effects

The difference between invalid-cue and valid-cue conditions were calculated as an index of validity effects (i.e., reorienting). See Supplementary Table S1 for the mean and SD of RT and accuracy in the two groups. For RT, a 2 (*group*) × 2 (*VF*) mixed ANOVA revealed that all the *group, VF*, and the interaction effects were not significant [*F*(1,53) < 1.539, *p* > 0.22, η_*p*_^2^ < 0.028; see Figure 3B]. For accuracy, the *VF* effect was significant [*F*(1,53) = 25.265, *p* < 0.001, η_*p*_^2^ = 0.323; see Figure 3E]; the *group* effect was significant [*F*(1,53) = 4.955, *p* = 0.03, η_*p*_^2^ = 0.085]; the interaction effect was also significant [*F*(1,53) = 10.597, *p* = 0.002, η_*p*_^2^ = 0.167]. Simple effects analyses with Bonferroni correction revealed that there was no significant difference between the left and right VF reorienting in AVGPs [*F*(1,53) = 1.659, *p* = 0.203, η_*p*_^2^ = 0.03; see Figure 3B]. But the validity score of NAVGPs was higher in the right than left VF [*F*(1,53) = 32.52, *p* < 0.001, η_*p*_^2^ = 0.38; see Figure 3E]. Furthermore, the validity scores of the right VF were significantly higher in NAVGPs than in AVGPs [*F*(1,53) = 10.587, *p* = 0.002, η_*p*_^2^ = 0.167], but there was no significant difference between the two groups in the validity scores of the left VF [*F*(1,53) = 0.478, *p* = 0.493, η_*p*_^2^ = 0.009; see Figure 3E].

### Disengaging Effects

The difference between invalid-cue and center-cue conditions were calculated as an index of disengaging effects. See Supplementary Table S1 for the mean and SD of RT and accuracy in the two groups. For RT, a 2 (*group*) × 2 (*VF*) mixed ANOVA revealed all the main or interaction effects were not significant [*F*(1,53) < 2.44, *p* > 0.124, η_*p*_^2^ < 0.044; see Figure 3C]. For accuracy, a 2 (*group*) × 2 (*VF*) mixed ANOVA revealed that the *VF* effect was significant [*F*(1,53) = 20.67, *p* < 0.001, η_*p*_^2^ = 0.281; see Figure 3F], indicating a left-VF bias for disengaging. The *group* effect was marginally significant [*F*(1,53) = 3.914, *p* = 0.053, η_*p*_^2^ = 0.069], indicating that the disengaging of AVGPs was more efficient than that of NAVGPs. Furthermore, the interaction effect was also significant [*F*(1,53) = 17.028, *p* < 0.001, η_*p*_^2^ = 0.243]. Simple effects analyses with Bonferroni correction revealed that there was no significant difference between the left and right VFs in AVGPs [*F*(1,53) = 0.093, *p* = 0.761, η_*p*_^2^ = 0.002; see Figure 3F]. However, NAVGPs showed a higher disengaging in the right than left VF [*F*(1,53) = 35.664, *p* < 0.001, η_*p*_^2^ = 0.402; see Figure 3F]. Furthermore, the right-VF disengaging scores were significantly higher for NAVGPs than AVGPs [*F*(1,53) = 12.077, *p* < 0.001, η_*p*_^2^ = 0.186], but there was no significant difference between the two groups for the left-VF disengaging [*F*(1,53) = 0.005, *p* = 0.944, η_*p*_^2^ = 0; see Figure 3F].

### Interactions Between Factors

A 4 × 2 × 2 × 2 ANOVA with *cue type* (no-cue, center-cue, valid-cue, and invalid-cue), *congruency* (congruent, incongruent), *VF* (left, right), and *group* (AVGPs, NAVGPs) as factors was conducted to extract interactions involving *group* (see Supplementary Table S2 for the details of the main and interaction effects). Significant interactions involving *group* were only found in the accuracy data. The *cue by group* interaction was significant [*F*(3,159) = 3.492, *p* = 0.017, η_*p*_^2^ = 0.062]. Simple effects analysis further revealed that AVGPs were significantly higher than NAVGPs only in the invalid-cue condition [*F*(1,53) = 4.207, *p* = 0.045, η_*p*_^2^ = 0.074] but not in the other conditions [*F*(1,53) < 0.905, *p* > 0.341, η_*p*_^2^ < 0.017].

**FIGURE 4:**
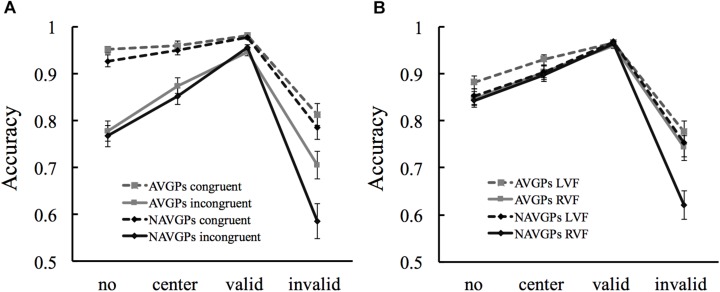
Simple effects analyses for the *cue by congruency by group* interaction **(A)** and the *cue by VF by group* interaction **(B)**. L/RVF, left/right visual field. Error bars denote SEM.

It was also found that the *cue by congruency by group* and *cue by VF by group* interactions were significant [*F*(3,159) > 3.978, *p* < 0.009, η_*p*_^2^ > 0.07]. The *cue by congruency* and *cue by VF interactions* were then conducted for each *group* to detail the significance of the interactions. The *cue by congruency* interaction was significant in both AVGPs [*F*(3,84) = 12.763, *p* < 0.001, η_*p*_^2^ = 0.313] and NAVGPs [*F*(3,75) = 14.913, *p* < 0.001, η_*p*_^2^ = 0.374]. Interestingly, the differences in accuracy between the congruent and incongruent conditions decreased from no/center/valid-cue to invalid-cue for both groups, but the decrease was larger for NAVGPs than AVGPs (Figure 4A). The *cue by VF* interaction was not significant in AVGPs [*F*(3,84) = 1.082, *p* = 0.361, η_*p*_^2^ = 0.037], but significant in NAVGPs [*F*(3,75) = 20.002, *p* < 0.001, η_*p*_^2^ = 0.444]. Specifically, the change in accuracy from no/center/valid-cue to invalid-cue conditions was smaller for AVGPs than NAVGPs when the cues were presented in the right VF, and the change in accuracy from the left to right VF was also smaller for AVGPs than NAVGPs when the cues changed from no/center/valid-cue to invalid cue (Figure 4B). As shown in Figure 4, the significance of the two interactions was mainly attributed to the invalid-cue condition.

## Discussion

The present study aimed to examine the lateralization and efficiency of attentional components, and their associations with AVG playing. We found that different from NAVGPs showing a right-hemisphere lateralized pattern for executive and reorienting components, AVGPs showed a bilateral mode (Figures 2, 3). However, alerting component was comparable in the two groups, not showing a lateralized pattern. The findings are in line with a recent study wherein a reduced leftward bias of visuospatial attention in AVGPs was revealed (Latham et al., 2014). It has been reported that the alerting component is bilaterally implemented in the brain (e.g., Greene et al., 2008; Asanowicz et al., 2012; Spagna et al., 2016, 2018), which gets supported by both the RT and accuracy results of alerting in NAVGPs. The absence of the differences between AVGPs and NAVGPs is consistent with a previous study showing no significant differences between the two groups for both children and adults (Dye et al., 2009). The finding that alerting was also comparable for the two groups in terms of lateralization suggests that AVG playing is not associated with alerting functions.

Previous studies have found that AVGPs have a more efficient executive function for resolving or suppressing cognitive conflicts as shown in different tasks compared to NAVGPs, (Chisholm et al., 2010; Mishra et al., 2011). Moreover, studies using the LANT revealed that executive functions show a bias to the right hemisphere in the general population (Greene et al., 2008; Asanowicz et al., 2012; Marzecová et al., 2013; Spagna et al., 2018), echoing the literature (Kondo et al., 2004; Levy and Wagner, 2011; Vallesi, 2012; Aron et al., 2014; Cai et al., 2014). The current study confirmed this finding. However, executive functions were comparable for both hemispheres of AVGPs, indicating a strong link between the bilateral executive functions and AVG playing. The bilateral executive functions of AVGPs likely reflects that neural resources are bilaterally recruited to detect and resolve cognitive conflict occurring in both VFs more efficiently. There were no significant differences between the left and right VFs of AVGPs, and no significant differences in the left VF between AVGPs and NAVGPs. These findings suggest that AVG playing is tightly associated with the efficiency of executive functions in the left VF such that executive functions could be performed comparably and unbiasedly for both VFs. Interestingly, a recent study found a larger conflict effect in AVGPs than in NAVGPs (Dye et al., 2009), but we did not (Figure 2D and Supplementary Table S2). Detailed analysis of Dye et al.’s data revealed that AVGPs responded more slowly to the incongruent flankers compared to the congruent flankers, leading to a larger conflict effect in AVGPs. This pattern was not observed in both RT and accuracy of the current study. The differences in results could be due to the age differences and the types of tasks in the studies. Dye et al. (2009) examined children aged 7–13 years using a child version of ANT, whereas our participants were adults who did a lateralized ANT.

The accuracy data of validity effects indicates that reorienting is dominantly supported by the right hemisphere. More specifically, the responses to stimuli preceded by invalid-cues were more accurate when stimuli were presented in the left VF than in the right VF. It concurs with several studies using the LANT (Greene et al., 2008; Asanowicz et al., 2012; Spagna et al., 2018). In this study, AVGPs showed a more efficient reorienting than NAVGPs; and AVGPs showed a bilateral reorienting function, but NAVGPs showed a reorienting biased to the right hemisphere (Figures 3E,F). It has been found that reorienting is dominantly implemented in the right hemisphere covering the temporoparietal junction and middle frontal cortex (Vossel et al., 2006; Petersen and Posner, 2012; Geng and Vossel, 2013; Krall et al., 2015). Therefore, the absence of the lateralization of reorienting in AVGPs exactly reflects the association of the improvement of the left hemisphere neural resources for reorienting functions with game playing, and the improvement could erase the differences in performance between hemispheres. It was proposed that reorienting is realized by the interplay between the dorsal system area, the intraparietal sulcus, and the ventral system area, the temporoparietal junction (Corbetta et al., 2008), so it is possible that AVGPs have more balanced interplays between the two systems of attention. Similar to the pattern of reorienting, AVGPs also showed a bilateral disengaging function while NAVGPs showed a disengaging function that is biased to the right hemisphere (Figures 3E,F). Both reorienting and disengaging reflects the capacity of responding to novel, unexpected but behaviorally relevant stimuli (Posner, 1980). The results of reorienting and disengaging collectively suggest a close relationship between game playing and the efficiency of capturing unexpected but behaviorally relevant stimuli.

There were no significant differences in orienting effects between the left and right VFs in both groups, indicating that game playing may not be related to hemisphere lateralization when the task is to capture cued, but always expected stimuli. Interestingly, AVGPs had faster responses to targets preceded by center-cues compared to NAVGPs, but the two groups benefited similarly from valid-cues, explaining why AVGPs had a lower score of orienting. The finding demonstrates the importance of separately analyzing the two conditions in interpreting orienting effects (Dye et al., 2009). The insignificant difference in only the valid-cue condition between the two groups is consistent with previous studies (Castel et al., 2005; Hubert-Wallander et al., 2011). However, using a temporal order judgment task West et al. (2008) found that AVGPs were more sensitive to early exogenous cues than NAVGPs, being opposite to the current finding. The reason could be that subjective report of temporal order judgment used in West et al. (2008) study might be much modulated by top-down attention rather than pure exogenous cue (Hubert-Wallander et al., 2011).

Unveiling how factors such as congruency, cue type, and VF interact with each other helps to better understand how the brain optimizes the processing of behaviorally relevant information (Callejas et al., 2004, 2005; Fan et al., 2009; Badre, 2011; Marotta et al., 2012; Xuan et al., 2016; Spagna et al., 2018). The finding that AVGPs had more efficient interplays between congruency or VF and cue type for a better attentional capacity (Figure 4) suggest that AVGPs are less susceptible to the factors, congruency, VF, and cue type. The results of the *cue by congruency by group* interaction is likely to indicate that NAVGPs’ conflict resolving is not efficient during reorienting (Corbetta et al., 2008; Figure 4A). This is consistent with previous studies (e.g., Fan et al., 2009; Trautwein et al., 2016). However, AVGPs have optimized interplays between reorienting and conflict resolving such that conflicts could be efficiently resolved during reorienting to targets (Figure 4A), possibly reflecting the important role of anterior insula cortex in the interplays (Trautwein et al., 2016). The findings provide strong evidence for the association between AVG playing and the enhancement of the interplays between different factors.

Note that the enhanced attentional components of AVGPs observed in this study could be attributed to factors such as enhanced ability to “learning to learn” (Bejjanki et al., 2014; Green and Bavelier, 2015), better visuospatial resolution (Green and Bavelier, 2007), or larger useful field of view (Green and Bavelier, 2003, 2006; Feng et al., 2007). For example, Bejjanki et al. (2014) found that AVGPs and NAVGPs had similar performance on the earliest trials of a new task, but gamers showed steeper learning functions. Therefore, it is possible that the differences in the ability to “learning to learn” between the two groups contributed to the differences in performance, although participants knew little about the ratio of valid- and invalid-cue trials. It is also possible that AVGPs’ larger useful field of view and lower threshold of visual resolution led to better performance, especially in detecting targets preceded by invalid-cues and resolving conflicts evoked by incongruent flankers.

The human brain is highly plastic. Reduced lateralization of visuospatial attention has been found in musicians (e.g., Patston et al., 2006, 2007) and bilinguals especially for executive functions (e.g., Marzecová et al., 2013), clearly indicating learning a second language and performing music instruments may reshape the lateralization of the human attentional networks. Extending the existing literature showing AVGPs have better attentional abilities (Green and Bavelier, 2012), the current study revealed that the lateralization of attentional networks is highly associated with AVG playing, contributing to a better understanding of improvement in attentional capacity, and more generally, neural plasticity benefiting from extensive game playing. However, the results do not indicate a causal relationship between AVG playing and the changes of attentional network. Future studies can take advantage of randomized control trial to examine how AVG playing changes or reshapes attentional networks, by which a causal inference can be made.

## Conclusion

Using the LANT, we found that unlike NAVGPs possessing executive and reorienting functions being biased to the right hemisphere, the two were bilaterally implemented in the brain of AVGPs. But alerting functions were comparable for both populations in terms of lateralization. The results indicate that AVG playing is associated with the efficiency and lateralization of attentional networks, especially for executive and reorienting functions, and with overall reduced lateralization of visuospatial attention.

## Data Availability

All datasets generated for this study are included in the manuscript and/or the Supplementary Files.

## Ethics Statement

This study was approved by the Institutional Review Boards of Qufu Normal University and each participant signed an informed consent form before the experiment.

## Author Contributions

YL and DN designed the study and drafted the manuscript. XJ and YW collected the data. YL and XJ analyzed the data.

## Conflict of Interest Statement

The authors declare that the research was conducted in the absence of any commercial or financial relationships that could be construed as a potential conflict of interest.
