# Supplementary material for: Reduced Lateralization of Attention in Action Video Game Players
Source: Front Psychol. 2019 Jul 17;10:1631. doi: 10.3389/fpsyg.2019.01631 (PMC6650590; doi:10.3389/fpsyg.2019.01631)
Supplement: Supplementary file 1 [file Data_Sheet_1.docx]

**Supplementary materials**

*
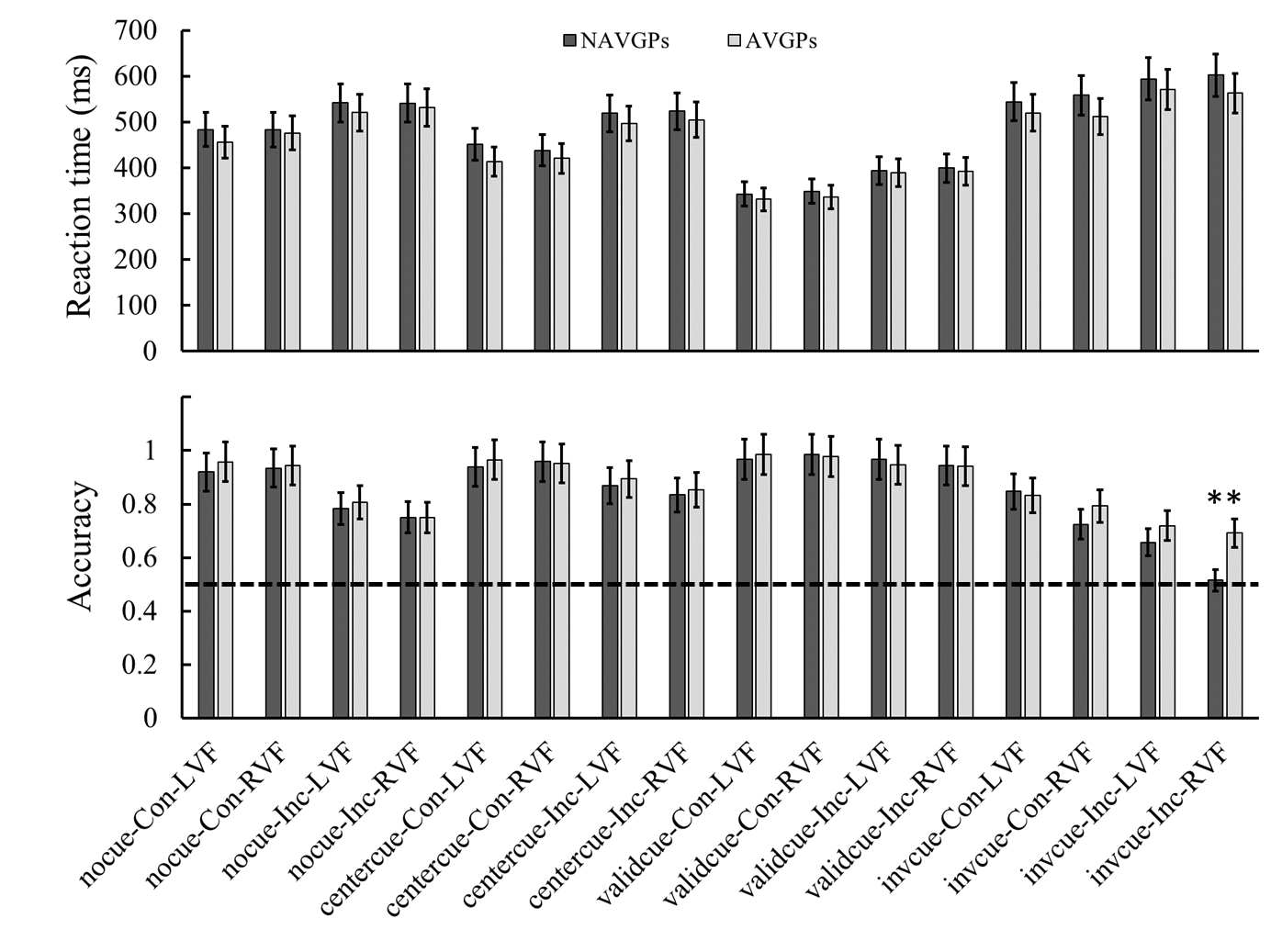
Figure S1*. Reaction time (ms) and accuracy for each condition in the non-action video game players (NAVGPs) and action video game players (AVGPs). The dashed line denotes the chance level (.5). ** *p* < 0.01.

Table S1. Mean and SD of reaction time (ms) and accuracy for each effect in the non-action video game players (NAVGPs) and action video game players (AVGPs).

|  |  | AVGPs | |  | NAVGPs | |
| --- | --- | --- | --- | --- | --- | --- |
|  |  | Mean | SD |  | Mean | SD |
| Reaction time | Alerting LVF | 33 | 35 |  | 27 | 38 |
|  | Alerting RVF | 41 | 40 |  | 31 | 34 |
|  | Executive LVF | 64 | 29 |  | 57 | 35 |
|  | Executive RVF | 62 | 35 |  | 59 | 37 |
|  | Orienting LVF | 95 | 35 |  | 117 | 35 |
|  | Orienting RVF | 98 | 38 |  | 107 | 20 |
|  | Validity LVF | 185 | 78 |  | 201 | 90 |
|  | Validity RVF | 172 | 65 |  | 206 | 96 |
|  | Disangaging LVF | 90 | 67 |  | 84 | 67 |
|  | Disangaging RVF | 74 | 62 |  | 100 | 94 |
| Accuracy | Alerting LVF | 0.05 | 0.07 |  | 0.05 | 0.07 |
|  | Alerting RVF | 0.06 | 0.08 |  | 0.05 | 0.06 |
|  | Executive LVF | 0.09 | 0.07 |  | 0.10 | 0.08 |
|  | Executive RVF | 0.11 | 0.08 |  | 0.14 | 0.09 |
|  | Orienting LVF | 0.04 | 0.05 |  | 0.06 | 0.06 |
|  | Orienting RVF | 0.06 | 0.07 |  | 0.07 | 0.06 |
|  | Validity LVF | 0.19 | 0.12 |  | 0.22 | 0.14 |
|  | Validity RVF | 0.22 | 0.13 |  | 0.35 | 0.16 |
|  | Disangaging LVF | 0.15 | 0.11 |  | 0.15 | 0.12 |
|  | Disangaging RVF | 0.16 | 0.12 |  | 0.28 | 0.13 |

Table S2. Results of the omnibus ANOVA.

|  |  | **reaction time** | | |  | **accuracy** | | |
| --- | --- | --- | --- | --- | --- | --- | --- | --- |
| **Effects** | ***df*** | ***F*** | ***p*** | ***η_p_^2^*** |  | ***F*** | ***p*** | ***η_p_^2^*** |
| *cue* | 3 | 263.161 | .001 | .832 |  | 139.856 | .001 | .725 |
| *con* | 1 | 248.275 | .001 | .824 |  | 155.441 | .001 | .746 |
| *VF* | 1 | 1.600 | .211 | .029 |  | 20.829 | .001 | .282 |
| *group* | 1 | .615 | .436 | .011 |  | 2.687 | .107 | .048 |
| *cue * group* | 3 | 1.182 | .318 | .022 |  | 3.492 | .017 | .062 |
| *con * group* | 1 | .381 | .540 | .007 |  | 1.116 | .296 | .021 |
| *VF * group* | 1 | .097 | .756 | .002 |  | .793 | .377 | .015 |
| *cue * con* | 3 | 9.762 | .001 | .156 |  | 24.820 | .001 | .319 |
| *cue * VF* | 3 | .258 | .856 | .005 |  | 13.595 | .001 | .204 |
| *con * VF* | 1 | .003 | .957 | .000 |  | 5.465 | .023 | .093 |
| *cue * con * group* | 3 | .072 | .975 | .001 |  | 3.978 | .009 | .070 |
| *cue * VF * group* | 3 | 1.903 | .131 | .035 |  | 9.371 | .001 | .150 |
| *con * VF * group* | 1 | .251 | .618 | .005 |  | 1.361 | .249 | .025 |
| *cue * con * VF* | 3 | .308 | .820 | .006 |  | .774 | .510 | .014 |
| *cue * con * VF * group* | 3 | .212 | .888 | .004 |  | .120 | .948 | .002 |

Note: VF, visual field; con, congruency; significant effects are highlighted in blue.
